# Occupational and leisure time physical inactivity and the risk of type II diabetes and hypertension among Mexican adults: A prospective cohort study

**DOI:** 10.1038/s41598-018-23553-6

**Published:** 2018-03-29

**Authors:** C. Medina, I. Janssen, S. Barquera, S. Bautista-Arredondo, M. E. González, C. González

**Affiliations:** 1 Mexican National Institute of Public Health, Health and Nutrition Research Center, Cuernavaca, Mexico; 2 Queen’s University, School of Kinesiology and Health Studies, Kingston, Canada; 3 Mexican National Institute of Public Health, Division of Health Economics, Cuernavaca, Mexico; 4 Mexican National Institute of Public Health, Unit for Research in Diabetes and Cardiovascular Risk, Cuernavaca, Mexico; 5 Universidad Nacional Autónoma de México, Mexico City, Mexico; 6 Centro de Estudios en Diabetes, Mexico City, Mexico

## Abstract

There is a lack of longitudinal data linking physical inactivity and chronic diseases among Mexicans. *Objective*. To examine the relationship between total, leisure and occupational moderate-to-vigorous physical activity (MVPA) and incidence of type II diabetes (T2D) and hypertension in the Mexico City Diabetes Study. *Study design and population*. A prospective cohort study was conducted from 1989 to 2009 among 2282 men and non-pregnant women residing in six low-income neighborhoods in Mexico City. *Main outcome*. Incidence of T2D and hypertension. *Results*. After controlling for confounders, <1 MET/min/week of MVPA during leisure time was associated with higher risk of hypertension (HR 1.29, CI 95% 1.01, 1.66) and T2D (HR 1.31 CI 95% 1.00, 1.74). In addition, accumulating <1 MET/min/week of occupational MVPA was associated with higher risk of hypertension (HR 1.47, CI 95% 1.13, 1.90). *Conclusion*. The absence of leisure and occupational MVPA was associated with an increased risk of hypertension. However, no associations were found between occupational MVPA and T2D.

## Introduction

Chronic diseases, mainly cardiovascular disease, cancer and diabetes, are the largest causes of death worldwide^1^. More than 80% of these deaths occur in low and middle income countries^1^. Within Mexico, a middle-income country, 31.5% of adults have hypertension^2^ and 14.4% have type II diabetes (T2D)^3^. Chronic diseases have major adverse effects on the quality of life and are an important contributors to premature deaths, and health care costs^1^. The epidemic of chronic diseases within low and middle income countries, such as Mexico, is largely attributable to changes in the food environment and lifestyles, including decreasing levels of physical activity^1^.

Several studies have demonstrated that physical inactivity is an important modifiable risk factor for hypertension^4^ and T2D^4,5^. Recent systematic reviews of intervention studies suggest that total physical activity reduces the incidence of hypertension and T2D by 34%^6^ and 42%^7^, respectively. Most of the studies analyzing the relationship between physical activity, hypertension, and T2D examined either leisure or total physical activity, and the few studies that examined occupational activity reported inconsistent results for hypertension^6,8–11^ and an inverse association for T2D^12^. In addition, most of the available evidence is based on populations living in high-income countries such as the United States, Japan, Finland, and Canada^7^. There is a lack of information on the relationships between total, leisure and occupational physical inactivity and chronic diseases in middle-income Latin American countries such as Mexico. Therefore, the objective of this study was to examine the relationship between total, leisure and occupational physical activity and the incidence of hypertension and T2D within Mexican adults.

## Methods

### Study design and population

Our data was obtained from the *Mexico City Diabetes Study*, a prospective cohort study that started in 1989 with the selection of low-income areas in Mexico City. The low-income areas encompass six census tracts defined by the National Institute of Geography and Statistics (Instituto Nacional de Estadística y Geografía). From 15,532 inhabitants living in this area, all men, women and non-pregnant aged 35–64 years were selected. From a total of 3,505 individuals, 2,282 were followed for 20 years. The main purpose of the *Mexico City Diabetes Study* was to characterize the prevalence, incidence and natural history of T2D and cardiovascular disease risk factors^13,14^. Physical examinations and questionnaires on this cohort were collected at different time periods. Examinations consisted on anthropometric measurements (height, weight, waist and hip circumferences, skinfolds), blood pressure, oral glucose tolerance test (using a 75 gr glucose load), and a fasting blood sample through physical examinations and questionnaires. The questionnaire contained items that assessed sociodemographic characteristics, the history of diagnosed medical conditions (including T2D and hypertension), and medication use that were collected in 4 different time periods (1989–1990, 1993–1994, 1997–1998, 2008–2009). Lifestyle behaviors (alcohol, smoking, diet, physical activity) were collected only at baseline (1989–1990)^15^. Death certificates or verbal autopsies were used to identify the date and cause of death among cohort participants who died during the follow-up period.

### Physical Activity Exposure Variables

The exposure variable was the average minutes per week of moderate, vigorous and very vigorous physical activity performed at work and leisure time. Physical activity levels were measured at baseline (1989–1990) using the Stanford Seven-day physical activity recall^16^. This recall requests participants to report cumulative sleep, moderate, vigorous and very vigorous activities for the previous 7 days. This instrument has been validated in several countries and it has been shown to have good reliability for moderate (r = 0.75) and vigorous (r = 0.83)^16^ activities and a modest validity (r = 0.54) for total physical activity^17^. For both domains (work and leisure) moderate, vigorous and very vigorous minutes/week were converted to Metabolic Equivalent Task (MET)/minutes/week by multiplying total minutes/week by 4, 6, and 10 respectively^16^. Moderate, vigorous and very vigorous METs per week were summed to create total MET/minutes/week of moderate-to-vigorous physical activity (MVPA) for work and leisure time. Work and leisure MVPA MET/minutes/week were summed to create total MVPA. Finally, MET/minutes/week for leisure, work and total MVPA were categorized in four groups: (1) <1 MET/minutes/week, (2) 1-599.9 MET/minutes/week, (3) 600-1199.9 MET/minutes/week, and (4) 1200 or more MET/minutes/week. These MET/minutes/week cut points were selected to represent no MVPA, a volume of MVPA that fell below the physical activity guidelines, a volume of MPVA that exceeded the physical activity guidelines, and a volume of physical activity that was at least double the physical activity guidelines^18^.

### Hypertension Outcome Variable

Trained nurses measured blood pressure at baseline and in each subsequent examination using a random zero mercury sphygmomanometer (Hawksley). Participants rested for 5 minutes before having their blood pressure measured 3 times. The average blood pressure from measurements 2 and 3 was used for analyses. Participants were classified as hypertensive if their blood pressure was equal or greater than 140/90^19^, self-report of physician diagnosed hypertension, if they were taking anti hypertensive medication, or (for deceased participants only) hypertension was listed as a cause of death on the death certificate. Cohort members with prevalent hypertension at the baseline exam, a history of myocardial infarction or stroke at baseline, or who reported high blood pressure during pregnancy at baseline were excluded from the hypertension analyses.

### T2D Outcome Variable

T2D was defined as a fasting glucose ≥7.0 mm/l (≥126 mg/dl), 2-hr post oral glucose tolerance test glucose of ≥11.1 mm/l (200 mg/dl)^20^, self-report of physician diagnosed T2D, taking diabetes medications (insulin or oral antidiabetic agents), or (for deceased participants only) diabetes was listed as a cause of death on their death certificate. Cohort members with prevalent T2D at the baseline exam, a history of myocardial infarction or stroke at baseline, and those who reported high levels of glucose during pregnancy at baseline were excluded from the T2D analyses.

### Covariates

Several sociodemographic (sex, age, educational level, marital status, family history of T2D), lifestyle (current smoking, total energy intake, sleeping hours and alcohol consumption) and anthropometric (body mass index) factors at baseline were considered. Education level was stratified into three groups according to the highest level of education obtained: primary or less, secondary, and high school or higher. Marital status was classified as married (legal marriage or common law) or not married (widow, divorced, separated, or single). Family history of T2D was classified as “yes” or “no” to the question: Do your parents have T2D? BMI was calculated from measured heights and weights^21^. Current smoking status (baseline) was obtained by asking: Do you smoke cigarettes? Alcohol intake was classified as “no” (participants that did not consume any alcohol in a typical week) or “yes” (those that consumed at least 1 gr per week). Total energy intake was estimated by a one-year food frequency questionnaire that lists 36 common foods from Mexico City^22^. Sleeping hours were obtained by asking: On average, how many hours did you sleep on the day before going to work or the day before (if participants do not work)?

To minimize the loss of participants because of missing values, missing covariate data was replaced with an imputed value. The SPSS imputation function was used to replace continuous (BMI, total energy intake, and sleeping hours) and categorical (marital status, education level, current smoking, and history of T2D) missing variables. Multiple imputation involved 4 steps: 1) analyzing the missing patterns of each variable, 2) creating five data sets with imputed values, 3) conducting the analysis on each of the five data sets, and 4) reporting the result of the 5 pooled data sets. The overall percentage of missing data was: 5.5% for marital status, 0.4% for educational level, 0.3% for current smoking, 2.5% for family history of T2D, 0.1% for BMI, 0.0001% for total energy intake, and 0.5% for sleep hours. For the hypertension analyses, 6.7% of the cohort had at least one missing covariate and for T2D 8.2% had at least on missing covariate.

### Follow-Up Length/Time to Survival

The duration of follow-up was calculated as the time between the 1989–90 (baseline examination date) and whichever of the following came first: the date of confirmed T2D or hypertension, the date of death from any cause, the date the participant was lost to follow-up, or 2009 (end of the study period). The date of confirmed T2D or hypertension was set as either the date of the clinical examinations in this study, the date of death for any of these causes, or the date of physician diagnosis reported by the participants (if participants could not remember the exact day and month the 1^st^ day and first month was imputed)^13–15^. For T2D, 5.5% (1% day and 4.6% day and month) of the dates were imputed and for hypertension 12.1% (2.7% day and 9.5% day and month) of the dates were imputed.

### Statistical analysis

Analyses were performed using SPSS software version 24. Standard descriptive statistics were used to characterize the cohort. Incidence rate was obtained by dividing the number of cases by person-years of follow-up in each physical activity category. The incidence rates were age-standardized by the direct method using the total population as the standard^23^. Age groups were categorized as 30–34, 35–39, 40–44, 45–49, 50–54, 55–59, 60–64 and 65–69. Complex Sample Cox Regression model was used to estimate hazard ratios for hypertension and T2D (dependent variables) according to physical activity (independent variable) at baseline adjusted for age (Model 1). Age-adjusted variable was introduced into the model as a weight sample variable in order to control for the effect of age among years. In addition, cluster and strata variables were introduced as constant variables. Low-income (=1) was introduced as strata. Cluster was the ID number in the cohort study (from 1 to 2282). These variables were introduced into the model to indicate that there were no strata neither clusters in the selection of participants. Assumptions of the model (multicollinearity, log rank and differences between included and excluded participants) were tested before further analysis. Sensitivity analysis was performed using 4 different imputed dates (random day/month, random day, always the 15^th^ of month, always the first of the month). The association between diabetes and hypertension with the physical activity variables was the same in all four imputations (data not shown). Interactions between natural logarithm time and covariates for each health condition were tested using the Cox with time-dependent covariate function. Statistically significant interactions were considered in the models. The level of significance was set at p < 0.05. Model 2 was controlled for the covariates described above, both leisure and work MVPA, and for family history of T2D except for BMI. Model 3 was controlled for all covariates.

## Results

The characteristics of the cohort at baseline are shown in Table 1. Of the 2,282 participants, 342 (15%) had T2D and 651 (28.5%) had hypertension at baseline and were excluded from the follow-up analysis. Therefore, the prospective analysis was limited to 1,883 participants for T2D and 1,541 participants for hypertension. The median BMI was 27.5 kg/m^2^ for the diabetes sample and 26.9 kg/m^2^ for the hypertension sample. More than 70% of the cohort was classified within the lowest educational level. The median fasting glucose was 84.3 mg/dl for the diabetes sample and 85.1 mg/dl for the hypertension sample. The median of systolic and diastolic blood pressure was 114/72 mmHg for the diabetes sample and 112/71 mmHg for the hypertension sample. Participants reported higher levels of MVPA during work (1620 and 1500 MET min/week for diabetes and hypertension samples) than during leisure time (300 and 336 MET min/week for diabetes and hypertension samples).

**Table 1:** Baseline (1989–90) characteristics according to health condition in participants from the Mexico City Diabetes Cohort Study^a^.

Characteristic	Analytic sample for T2D	Analytic sample for hypertension
	N = 1883	N = 1541
Sex (% women)	58.3	54.9
Age, years (median, IQR)	45 (39, 52)	45 (39, 52)
Height, kg (median, IQR	156 (150, 163)	156.5 (150.2, 163.5)
Weight, kg (median, IQR)	68.2 (60.5, 76)	67.5 (59.7, 74.9)
BMI, kg/m^2^ (median, IQR)	27.5 (25.1, 30.3)	26.9 (24.7, 29.7)
Fasting glucose, mg/dl (median, IQR)	84.3 (76.5, 91.7)	85.1 (77, 94)
Systolic blood pressure, mmHg (median, IQR)	114 (105, 125)	112 (104, 120)
Diastolic blood pressure, mmHg (median, IQR)	72 (66, 79)	71 (65, 76)
**Education for sociodemographics**		
Primary or less (%)	73.6	72.6
Secondary (%)	16.1	16.4
High school or higher (%)	10.3	11.0
With couple (%)	87.1	87.9
Family history of diabetes (%)	23.5	25.5
Current smoking (%)	32.4	34.1
Energy intake, kcal/day (median, IQR)	2052 (1635, 2637)	2058 (1646, 2624)
Alcohol use (%)	33.6	35.4
Sleep duration, hr./day (median, IQR)	7.5 (6.5, 8)	7.5 (7, 8)
**Moderate-to-Vigorous Physical Activity**		
Occupational* MET/min/week (median, IQR)	1620 (380, 3160)	1500 (300, 3060)
Leisure* MET/min/week (median, IQR)	300 (0, 1350)	336 (0, 1353)

a- mutually exclusive samples.

For the T2D analyses, the median follow-up length was 14.4 person years (IQ range: 6.08–16.8 years), with a total of 21,835 person years of follow-up. For the hypertension analyses, the median follow-up length was 11.8 years (IQ range: 6.08–16.6 years), with a total of 16,666 person years of follow-up. There were 383 (17.2%) incident cases of T2D and 477 (31%) incident cases of hypertension. Based on the diabetes sample, the whole population was significantly younger, and had higher smoking and history of diabetes than missing participants. Based on the hypertension sample, the whole population was significantly younger than missing participants.

The association between leisure, occupational, and total MVPA with T2D is shown in Table 2. Participants that accumulated <1 METs/min/week of MVPA during their leisure time had 1.45 (95% CI: 1.10–1.92) higher risk of T2D by comparison to participants that achieved 1200 or more METs/min/week of MVPA (p = 0.008) after adjusting for covariates except BMI. This association remained significant after including BMI in the model 1.31 (95% CI: 1.00–1.74). No association was observed between occupational and total physical activity and T2D.

**Table 2:** Risk of T2D according to physical activity level.

Physical Activity Level (METs/min/week)	Total (N)	Incident Cases, n	Person-years of follow-up	Incidence rate per 1000 person years	Model 1^+^ HR (95% CI)	Model 2^∞^ HR (95% CI)	Model 3* HR (95% CI)
Occupational**							
1200 or more	861	232	12,785	18.1	1	1	1
600–1199.9	187	41	2,696	13.8	0.83 (0.59, 1.16)	0.91 (0.65, 1.28)	0.87 (0.62, 1.22)
1–599.99	253	59	3,290	17.9	1.01 (0.76, 1.35)	1.03 (0.77, 1.38)	0.93 (0.69, 1.24)
<1	240	51	3,063	16.6	0.95 (0.70, 1.30)	1.01 (0.74, 1.40)	0.98 (0.71, 1.35)
*P value for trend*					0.721	0.904	0.718
Leisure**							
1200 or more	442	95	6,352	14.9	1	1	1
600–1199.9	211	79	3,076	25.7	1.12 (0.80, 1.58)	1.18 (0.85, 1.66)	1.11 (0.79, 1.56)
1–599.99	323	50	4,316	11.6	1.29 (0.96, 1.74)	1.28 (0.95, 1.73)	1.11 (0.82, 1.50)
<1	565	156	8,089	19.2	**1.41 (1.08, 1.83)**	**1.45 (1.10, 1.92)**	**1.31 (1.00, 1.74)**
*P value for trend*					0.062	**0.008**	0.065
Total							
1200 or more	1168	299	17,031	17.5	1	1	1
600–1199.9	195	38	2,683	14.1	0.83 (0.59, 1.17)	0.82 (0.58, 1.15)	0.72 (0.51, 1.02)
1–599.99	137	37	1,631	22.7	**1.43 (1.01, 2.01)**	**1.42 (1.00, 2.00)**	1.15 (0.81, 1.63)
<1	41	9	488	18.4	1.30 (0.66, 2.55)	1.13 (0.58, 2.20)	1.13 (0.58, 2.22)
*P value for trend*					0.091	0.255	0.902

^+^Age adjusted.

^∞^Adjusted by sex, age, education levels, marital status, current smoking, alcohol intake, total energy intake, parent history of diabetes, sleeping hours and leisure/working METs/min/week. All variables at the baseline.

*Covariates used in model 2 and body mass index.

**Work and leisure included the sum of moderate, vigorous and very vigorous physical activity.

Table 3 contains the results of the analyses examining the association between physical activity and hypertension. After controlling for covariates except BMI, participants that accumulated <1 METs/min/week of leisure and occupational MVPA had increased risk of hypertension by comparison to those that achieved 1200 METs/min/week or more (HR 1.37, 95%CI: 1.07, 1.75, p = 0.015 and HR 1.52, 95% CI: 1.17, 1.97, p = 0.001) for leisure time and occupational activity, respectively). This association remained significant after including BMI into the model (HR 1.29 95%CI: 1.01, 1.66, p = 0.43 and HR 1.47, 95% CI: 1.13 1.90, p = 0.004) respectively. No association was observed between total physical activity and hypertension. A significant dose-response relationship was observed between leisure physical activity (p = 0.036) and occupational physical activity (p = 0.028) and the risk of hypertension.

**Table 3:** Risk of hypertension according to physical activity level.

Physical Activity Level (METs/min/week)	Total (N)	Incident Cases, n	Person-years of follow-up	Incidence rate per 1000 person years	Model 1^+^ HR (95% CI)	Model 2^∞^ HR (95% CI)	Model 3* HR (95% CI))
Occupational**							
1200 or more	1094	259	9,888	26.2	1	1	1
600–1199.9	227	67	2,054	32.6	1.28 (0.97, 1.70)	1.30 (0.99, 1.71)	1.29 (0.99, 1.70)
1–599.99	291	65	2,800	23.2	0.95 (0.72, 1.25)	0.97 (0.74, 1.28)	0.94 (0.71, 1.24)
<1	271	86	2,444	35.1	**1.41 (1.09, 1.83)**	**1.52 (1.17, 1.97)**	**1.47 (1.13, 1.90)**
*P value for trend*					**0.020**	**0.012**	**0.028**
Leisure**							
1200 or more	528	122	5,095	23.9	1	1	1
600–1199.9	255	67	2,537	26.4	1.09 (0.80, 1.47)	1.07 (0.79, 1.44)	1.02 (0.75, 1.37)
1–599.99	375	99	3,502	28.2	1.24 (0.94, 1.62)	1.19 (0.91, 1.56)	1.12 (0.86, 1.47)
<1	725	189	6,051	31.2	**1.39 (1.09, 1.76)**	**1.37 (1.07, 1.75)**	**1.29 (1.01, 1.66)**
*P value for trend*					**0.042**	**0.011**	**0.036**
Total							
1200 or more	1453	351	13,278	26.4	1	1	1
600–1199.9	226	72	2,178	33.0	**1.30 (1.01, 1.67)**	1.24 (0.96, 1.60)	1.20 (0.93, 1.55)
1–599.99	160	38	1,302	29.1	1.24 (0.88, 1.76)	1.13 (0.81, 1.59)	1.07 (0.76, 1.50)
<1	44	16	428	37.3	1.48 (0.85, 2.61)	1.50 (0.90, 2.49)	1.24 (0.74, 2.08)
*P value for trend*					0.086	0.055	0.235

^+^Age adjusted.

^∞^Adjusted by sex*T, age, education levels, marital status, current smoking, alcohol intake*T, total energy intake, sleeping hours and leisure/working METs/min/week. All variables at the baseline.

*Covariates used in model 2 and body mass index.

**Work and leisure included the sum of moderate, vigorous and very vigorous physical activity.

T = time (ln (T_).

## Discussion

This study examined the association between leisure, occupational and total METs/min/week MVPA and the risk of T2D and hypertension in a sample of low-income adults residing in Mexico City. Our findings indicate that absence of MVPA during leisure time and work was associated with a modestly increased risk of hypertension. However, the lack of occupational MVPA was not associated with T2D.

By comparison to adults in high-income countries, adults in low- and middle-income countries accumulate more MVPA during work^24,25^ and less MVPA during leisure time^24^. In our study of Mexican adults from poor neighborhoods of Mexico City, work was the main contributor to physical activity. Continuous surveillance of this pattern is needed, especially in developing countries, because according to the most recent evidence, occupational physical activity has decreased in low-income areas that are undergoing rapid socioeconomic development^24,26,27^. This phenomenon could be a contributor to the increasing trends on chronic diseases morbidity and mortality, including hypertension^28^ and T2D^27,29,30^.

Our study indicates that the absence of MVPA during work increases the risk of hypertension (by 47%) but not the risk of T2D. Although many studies have examined the association between physical activity and chronic diseases^31^, only few have focused on the effect of occupational physical activity and the results of these studies demonstrate an inverse association for diabetes^10,12^ and inconsistent outcomes for hypertension^6,8–11^. Some studies have demonstrated that occupational physical activity reduces the risk of hypertension^32^ while others have shown no association^9^ or even an increased risk of hypertension (in black men)^6^, cardiovascular diseases^11^, and all-cause mortality^33^. These discrepancies could be explained by the confounding effect of muscle mass on BMI among the most physically intense workers^34^, the minimal effect that occupational physical activity has on cardiorespiratory fitness^33^ the inflammatory process involved in the prolonged elevated heart rate during hard work^33,35^, and/or by chance. Further research is needed to clarify the impact that physically demanding jobs have on health^8^.

Not accumulating any MVPA during leisure time was associated with an ~30% increased risk of T2D and hypertension after controlling for potential confounders in our study. This result is consistent with previously published research and suggests that the effect of MVPA on the risk of diabetes and hypertension is similar irrespective of race/ethnicity and socioeconomic status^6,36–44^. The positive effect of physical activity on diabetes might be explained in part by an improved insulin sensitivity, acute increase in glucose uptake and delivery by the muscle cells via GLUT-4 transporters, structural changes in skeletal muscles (e.g., fiber size, and capillary density), an improved hormone regulation (e.g., cortisol), a better control on enzymes related to glucose and lipid metabolism (e.g., hexokinase, glycogen synthase, lipoprotein lipase) and the reduction of body weight and adiposity^41,45,46^. Physical activity could reduce blood pressure through a variety of mechanisms such as reducing the main risk factors for hypertension (e.g., body weight)^9,47^, reducing serum concentrations of cholesterol^48^, by improving insulin sensitivity and reducing hyperinsulinemia^47,48^ through the effects of insulin on the urinary sodium excretion^9,47^, and by attenuating the adrenergic sympathetic activity and the total peripheral resistance resulting in a reduction of arterial pressure^9,47^.

Our study has some limitations that must be carefully considered. We measured physical activity at one point in time during the baseline exam and this may not have represented habitual physical activity levels across the lifespan. Measurement error in self-report is inevitable, and the misclassification attributed to self-reported physical activity likely biased the association toward the null. In addition, social desirability and/or social approval could also affect self-reported physical activity. Diagnosis of hypertension and diabetes in our study was in part reliant on self-reported information, which may have also contributed to misclassification and underestimated associations. Although the models were adjusted for potential confounders, residual confounding remains as the consequences of the imperfect measurement of physical activity. The generalizability of our study is limited since the low-income areas of Mexico City have important differences in relation to other parts of the country in characteristics such as educational level and access to health services.

## Conclusions

We found that the lack of leisure and occupational MVPA was associated with an increased risk of hypertension in a low-income sample from Mexico City. However, no associations were found between occupational MVPA and T2D. Further research is needed to clarify the contribution of occupational MVPA on health, due to the fact that this domain is considered within the World Health Organization physical activity recommendations^18^.
